# Navigating a New Normal: A Mixed-Methods Study of the Pediatric Tracheostomy Parent-Caregiver Experience

**DOI:** 10.3390/children12070956

**Published:** 2025-07-21

**Authors:** Laine DiNoto, Adrianne Frankel, Taylor Wheaton, Desirae Smith, Kimberly Buholtz, Rita Dadiz, Kathryn Palumbo

**Affiliations:** 1 Department of Otolaryngology and Pediatrics, University of Rochester Medical Center, Rochester, NY 14642, USA; 2 Department of Pediatrics, University of Rochester Medical Center, Rochester, NY 14642, USA; adrianne_frankel@urmc.rochester.edu (A.F.); taylor_wheaton@urmc.rochester.edu (T.W.); desirae_smith@urmc.rochester.edu (D.S.); rita_dadiz@urmc.rochester.edu (R.D.); kathryn_palumbo@urmc.rochester.edu (K.P.); 3 School of Nursing, University of Rochester School of Nursing, Rochester, NY 14642, USA; kimberly_buholtz@urmc.rochester.edu

**Keywords:** children, complex medical care, tracheostomy, parent-caregiver

## Abstract

Objective: To explore the experiences and self-efficacy of parent-caregivers providing care for a child with a tracheostomy tube. Study Design: Parent-caregivers completed surveys and participated in semi-structured interviews about their experiences learning to care for their child with a tracheostomy tube. Survey data were analyzed using descriptive statistics. Interviews were transcribed verbatim and analyzed thematically through coding. Results: Fifteen parent-caregivers participated in the survey, 13 of whom completed an interview. After receiving a tracheostomy, children were hospitalized a median of 6 months prior to discharge home. At the time of our study, children had been home for a median of 3.5 years. Parent-caregivers felt more prepared to perform routine daily care compared to triaging a change in medical status. Parent-caregiver self-efficacy in performing tracheostomy care skills improved with experience at home. Four themes were identified from interviews: new identity formation, enduring education, child and family biopsychosocial support, and establishing normalcy. Parent-caregivers shared that education was more than just acquiring skills; it also involved discovering diverse ways of learning and building confidence in one’s own abilities to fulfill the many types of roles they serve to successfully care for and keep their child safe while supporting their social and emotional needs as parent-caregivers. Conclusions: Parent-caregivers’ reflections on their experiences provide critical insight into their psychosocial needs and challenges in providing care to children with tracheostomies. Further investigation of lived experiences is vital to shaping a community that can support families of medically complex children.

## 1. Introduction

In the United States, approximately 40,000 children have a tracheostomy as a lifesaving measure that allows for discharge from the hospital to either the home setting or a complex chronic care facility [1]. Children with a tracheostomy are reliant on medical technology to support airway or respiratory limitations and have increased healthcare needs [2]. Because of medical complexity, children with tracheostomies are at high risk for mortality, hospital readmissions, and significant morbidity, such as bleeding, tracheostomy tube dislodgement, and tracheostomy tube obstruction [3,4,5,6]. Parent-caregivers must learn all facets of caring for their child with a tracheostomy, which includes how to perform routine care, as well as respond to emergencies and adapt to life at home with a child who is reliant on technology. The American Thoracic Society and the American Academy of Head and Neck Surgery have published guidelines regarding caregiver tracheostomy education [1,7]. However, how education is implemented and what skills parent-caregivers need to demonstrate in the hospital varies among different institutions [8,9], which can affect parent-caregiver confidence and readiness to transition home. In addition, there remains a gap in understanding their lived experiences. Some studies have shown that parent-caregivers express stress during and after the hospital-to-home transition [10,11,12]. While the need for structured training is a main contributing factor [13], parent-caregivers also identify a lack of community resources that contribute to their anxiety [14,15]. Further identification of these and other factors is needed to better understand how to prepare, build confidence, and support parent-caregivers in the different facets of their child’s care in the home setting [16,17]. We therefore conducted a mixed-methods study of parent-caregivers who have been providing care to children with a tracheostomy in the home setting. We aimed to explore and identify factors from their lived experiences that both facilitated and posed challenges in their child’s transition from the hospital to their homes. The results of this study will contribute to the growing body of the literature to help guide changes in parent-caregiver education, as well as in hospital staff and home care nursing education, to better meet the needs of children with tracheostomies and their families.

## 2. Methods

### 2.1. Participants, Setting, Approval

In 2021–2022, we conducted a mixed-methods study consisting of surveys and interviews to assess parent-caregiver self-efficacy, in-hospital training, and experiences related to caring for a child with a tracheostomy tube. We identified parent-caregivers in this study to encompass those who are the primary caregivers for a child with a tracheostomy. Our use of the words “parent” and “caregiver” reflects the unique identities of these persons. A person may care for a child with a tracheostomy, for whom they love, provide for, and advocate on behalf of, but may not regard themselves as a parent. The use of the term “parent-caregiver” supports the psychosocial identity of such individuals [18].

Individuals eligible to participate in this study included parent-caregivers of children who received a tracheostomy tube between 2015 and 2020 at Golisano Children’s Hospital, a 170-bed, tertiary care hospital that is a part of the University of Rochester Medical Center. Located in Western New York with rural, suburban, and city areas, the children’s hospital serves the surrounding 17-county region with the only level I trauma center, pediatric cardiac surgical center, and level IV neonatal intensive care unit. We excluded parent-caregivers whose children received a tracheostomy tube at an outside institution, those who were not proficient in communicating in English during an interview, and those whose children were discharged from the hospital to a long-term care facility. This study was conducted according to the guidelines of the Declaration of Helsinki and was approved by the Institutional Review Board of the University of Rochester (STUDY00005759; 22 April 2021). Patient consent was waived due to no protected health information identified. The authors declare no conflicts of interest.

### 2.2. Survey: Design, Distribution, and Data Analysis

The survey was designed, piloted, and iteratively revised by a multidisciplinary team of experts in nursing, neonatology, otolaryngology, pediatric critical care medicine, and pulmonology by following a systematic approach to survey design [19]. Utilizing REDCap for distribution [20], the final survey consisted of 27 multiple-choice, Likert scale, and open-ended response questions. The survey captured two points in time: parent-caregivers’ level of preparedness at the time of initial discharge home from the hospital and their level of confidence in caring for their child at home at the time of the study. The survey focused on caregiver readiness to perform tracheostomy-related skills and self-efficacy in providing care to their child at home, as well as the challenges they experienced at home (Appendix A Table A1). The survey was distributed at a multidisciplinary tracheostomy clinic or sent securely via email or via the MyChart^©^ messaging application [21], an online patient portal in the electronic medical record. Participation was anonymous and voluntary. We utilized descriptive statistics to describe demographic data. Data are displayed as medians with interquartile ranges (Q1, Q3).

### 2.3. Interviews: Caregiver Participation, Interview Guide Design, and Data Analysis

We utilized convenience sampling to invite parent-caregivers who completed the survey to participate in individual semi-structured interviews utilizing Zoom video meetings [22]. The interview guide included sixteen open-ended questions designed to encourage parent-caregiver reflections on their preparation for caring for their child at home, their in-hospital training experience, any emergencies encountered at home, and their advice for other parent-caregivers and healthcare teams (Appendix A Table A2). We developed, iteratively revised, and piloted the interview guide with a caregiver who had a tracheostomy-dependent child (who was not eligible for this study) to ensure clarity and the relevance of questions. Investigators (AF, LD) co-facilitated interviews until reaching thematic saturation. Interviews were audio recorded, transcribed verbatim using Rev Transcription [23], and checked for accuracy.

The investigator team included an advanced practice provider (LD), nurses (KB, AF, DS), and physicians (RD, KP, TW) from neonatology (RD, DS), otolaryngology (LD), pediatric critical care medicine (AF, KP, TW), and nursing education (KB), who provide clinical care and educate parent-caregivers and hospital staff on tracheostomy care. Respiratory therapy was not involved in survey construction. At our institution, most tracheostomy education is conducted by nursing; respiratory therapy has a limited role in patient and family education. The investigator team developed the interview guide and conducted data analyses with the awareness that their experiences in training and supporting parent-caregivers may influence the study process. Their diversity in professional roles, location of work, and experiences in interacting with parent-caregivers added to the robustness of the discussion during data analysis.

Utilizing the principles of grounded theory, two investigators (AF, LD) coded each transcript independently and then discussed and refined the codes [24,25]. They applied codes to subsequent transcripts and refined them further as needed. If the investigators disagreed on a code, they involved a third investigator to reach an agreement (KP, TW, or RD). The third investigator reviewed all codes. All investigators participated in a series of immersion and crystallization cycles to draw contextual meaning, discuss themes and subthemes, and develop visual aids to conceptualize relationships [26,27]. To support the trustworthiness of data analyses, a fourth investigator reviewed the codes of 75% of transcripts and the audit trail of data analyses [25].

## 3. Results

**Figure 1:**
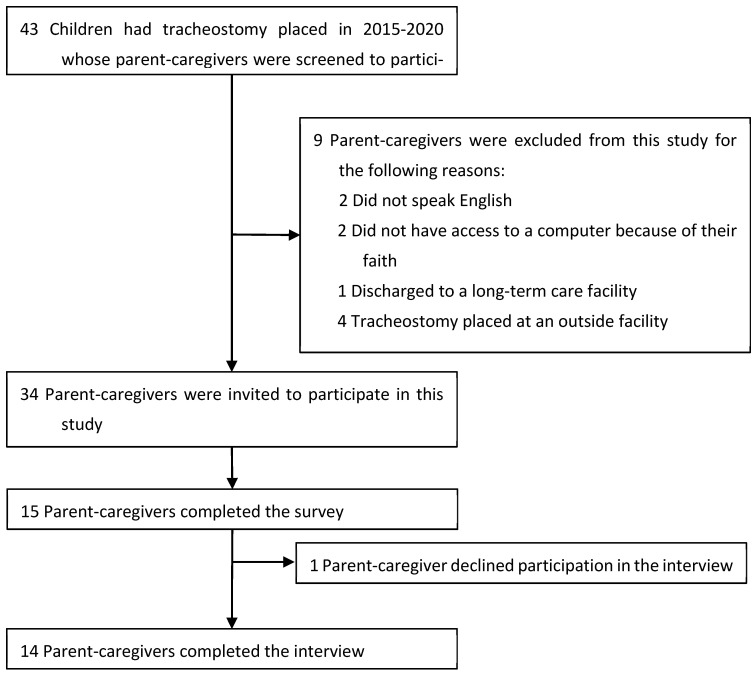
Inclusion and exclusion criteria flow diagram.

From the hospital’s pediatric tracheostomy database, we identified 43 children who had a tracheostomy tube placed between 2015 and 2020. Their caregivers were screened for eligibility to participate in this study (Figure 1). Of the 34 families who were eligible, 15 (44%) completed the survey, and 13 (38%) participated in an interview.

### 3.1. Survey

Parent-caregivers who participated in this study had children who obtained a tracheostomy at a median age of 4 (3, 6) months (Table 1). After the procedure, the children remained in the hospital for an additional 3 (2, 6) months for a total hospitalization of 6 (5, 10) months. When parent-caregivers participated in this study, the children had been at home with their caregivers for 42 (18, 54) months.

**Figure 2:**
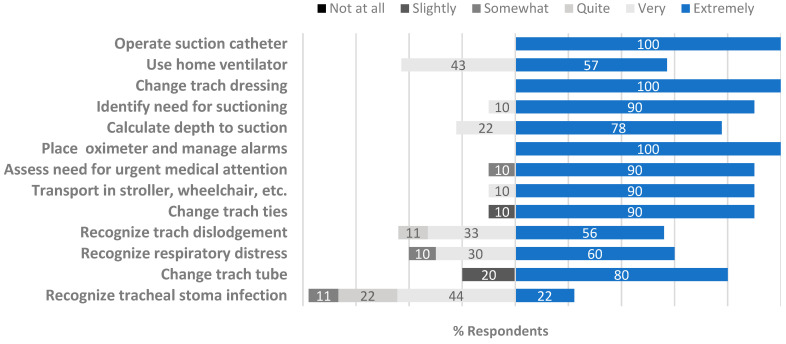
Confidence to perform tracheostomy skills at home (at time of study).

**Figure 3:**
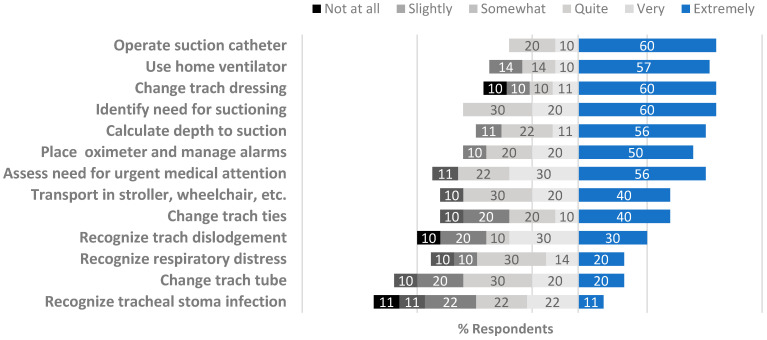
Preparedness to perform tracheostomy skills at home (at time of hospital discharge).

A total of 70% of children had their tracheostomy procedure while in the neonatal intensive care unit (NICU). Parent-caregiver education occurred in various locations of the children’s hospital, including the NICU, pediatric cardiac intensive care unit, pediatric intensive care unit, and pediatric general care units (Table 2). At the time of the survey, parent-caregivers reported that they were “very” or “extremely” confident to perform most tracheostomy skills (Figure 2). However, some parent-caregivers still felt “not at all” confident to change tracheostomy ties (5, (3, 6)) and change a tracheostomy tube (4 (3, 6)). Reflecting on their level of preparedness transitioning from the hospital to the home setting, parent-caregivers reported feeling “very” or “extremely” prepared to perform most tracheostomy skills (Figure 3). Parent-caregivers felt the least prepared to recognize tracheostomy tube dislodgement (6 (5, 6)), change a tracheostomy tube (6 (6, 6)), and recognize an infection (5 (4, 6)).

### 3.2. Interviews

We attained thematic saturation after conducting 13 interviews that averaged 54 ± 16 min. Participants included 12 mothers, 2 fathers, and 1 grandmother who served as primary caregivers. We identified four themes (italicized): *new identity formation*, *enduring education*, *biopsychosocial support*, and *establishing normalcy*. From these, 10 subthemes emerged (underlined in the paragraphs below). Table 3 presents interview excerpts that illustrate these themes and subthemes.

#### 3.2.1. Theme: New Identify Formation

Parent-caregivers shared the pivotal experience of a *new identity formation*. This occurred not only for them as a parent and a caregiver but also as a larger family unit. The theme is best illustrated with subthemes characterizing each type of role for which parent-caregivers learned new skills and responsibilities: caregiver, educator, employer, and entrepreneur. Each of these pillars has interdependent responsibilities that support and structure a new identity formation.

Parents expressed a divide between being a parent and being a caregiver. For some, the prior experience with their child was solely as a parent. Adapting to becoming a caregiver was vocalized as a different experience. For other parents, the first interaction with their child was as a caregiver, and then over time, they connected with being a parent (1 in Table 3). The experience of learning to be a caregiver was also identified as a process that was not necessarily inherent (2 in Table 3). Within these two distinct roles, there were conflicting demands between being a caregiver and being a parent, as well as tension with the ability to provide selfcare (3 in Table 3).

The second subtheme was becoming an educator. Initially, parent-caregivers identified themselves as learners. This included learning skills to care for their child. Parent-caregivers expressed they felt unprepared for the rapid transition from the role of learner to educator (4 in Table 3). Parent-caregivers became responsible for teaching nursing care specific to their child to private duty nurses (5 in Table 3). Many parent-caregivers expressed surprise at having to train private duty nurses themselves once their child was home.

In addition to taking on the role of an educator, parent-caregivers shared that they also became an employer and entrepreneur. To care for their child at home, many relied on private duty nursing, which required them to develop skills such as recruiting, conducting interviews, and hiring nurses from the community. These duties were often learned simultaneously while caring for their child in the hospital and trying to keep up with work and family obligations. Parent-caregivers posted advertisements, and flyers in the hospital and community, and joined different social media groups to recruit potential nurses. Parent-caregivers likened this process to that of a small business (6 in Table 3). Once home, parent-caregivers oversaw the “business” of their child: maintaining nursing schedules, submitting paperwork to insurance companies, and updating nursing orders and plans of care. Part of this management sector included scheduling medical appointments, maintaining and refilling medications, and ensuring home supplies were available, ordered, and delivered. All these responsibilities encompassed an entrepreneurial spirit, essentially running a startup business. Parent-caregivers shared the immense amount of time, organization, and extra resources it takes to have consistent care provided at home for their child (7 in Table 3). One family cited paying for a monthly faxing service for their nurses to submit insurance documentation and having to implement organizational strategies for their home care team (8 in Table 3). Another dedicated one full day per month to spend on the phone refilling prescriptions and obtaining prior authorizations. Each of these tasks demanded careful organization and focus.

#### 3.2.2. Theme: Enduring Education

*Enduring education* refers to the ongoing learning process that cumulates from the initial discussion about the need for a tracheostomy, through the procedure itself, the subsequent hospital course, and the eventual transition to the home environment. Enduring education encompasses three subthemes: experiential learning, confidence development, and parenting versus caregiving.

The first subtheme of *enduring education* was experiential learning. All parent-caregivers reported that the education they received in the hospital and the time spent practicing skills were beneficial in preparing for home. When asked about what information was the most helpful, parent-caregivers frequently cited reviewing and practicing emergency situations using low-fidelity tracheostomy dolls (9 in Table 3). When asked about using high-fidelity simulation in their training, interviewees identified this could have been helpful to increase confidence and competence while preparing for discharge. Parent-caregivers stressed the importance of hands-on practice. They also identified learning cardiopulmonary resuscitation for a medically complex child as a common and essential requirement (10 in Table 3). Some parent-caregivers sought out additional education on their own through other channels, including social media and online videos, to supplement their training in the hospital (11 in Table 3).

As parent-caregivers attained tracheostomy care skills, they gradually built confidence. This subtheme of confidence development was a unique experience for each family, though many parent-caregivers commented on the creation of a routine (12 in Table 3). The day-to-day life of providing care and triaging interventions for their child took time. Often parent-caregivers commented on the anxiety of their early days at home, which with time, evolved into a growing sense of confidence (13 in Table 3).

The third subtheme of enduring education was differentiating between caregiving versus parenting. For some families, these roles were learned and experienced simultaneously (14 in Table 3). Other families were parenting their children prior to the tracheostomy and learned over time how to navigate being a caregiver. One family found a lot of technical support in the hospital but felt there was a lack of education on parenting skills and child development (15 in Table 3).

#### 3.2.3. Theme: Biopsychosocial Support

Prolonged hospital stays with complex decision-making was a stressful experience. The tracheostomy decision was pivotal, and parent-caregivers wanted clear, balanced information. Parent-caregivers described the lack of peer support as challenging. One parent-caregiver expressed difficulty finding social support (16 in Table 3). In many communities and social circles, there were no other tracheostomy-dependent children (17 in Table 3). Some families rely on social media to connect with other parent-caregivers who have navigated life with a child with a tracheostomy.

The subtheme of compassion and respect refers to communication. It also encompasses a reflection on unconscious biases and value judgments. Parent-caregivers reported wishing that the hospital team was more aware and appreciative of stressors outside of the hospital setting. While their child is in the hospital, parent-caregivers are often challenged with balancing life at home, including the needs of other children, daily household responsibilities, and work obligations. They were appreciative of the care the hospital team provided, which enabled them to leave for periods of time and manage responsibilities at home. At the same time, parent-caregivers discussed a feeling of judgment and worried about how the medical team perceived them as parents when they needed to leave their child’s bedside. In addition, parent-caregivers poignantly identified each family’s different journeys, value systems, and backgrounds. Individual choices, reasons, and beliefs impacting the decision to pursue tracheostomy are unique. Parent-caregivers encouraged medical teams to reflect on these values (18 in Table 3). Some parent-caregivers also shared that the hospital team’s focus on the medical needs and diagnoses of their child overpowered their identity as their child (19 in Table 3).

#### 3.2.4. Theme: Establishing Normalcy

A final theme identified in this study was the sense of establishing normalcy. Families considered time itself a key factor in their development of confidence. Within the theme of establishing normalcy, the subthemes of simultaneous opposing emotions and routine prevailed as key features of the “new normal”.

Parent-caregivers experienced simultaneous opposing emotions when making the transition to home. It could be a joyous occasion that one looked forward to for a long time. Being home with their child after such a tremendous journey was met with relief (20 in Table 3). It could also be stressful as families learned how to navigate their child’s needs outside of the hospital. Returning to a life outside the hospital was difficult after lengthy hospital stays (21 in Table 3). Many parent-caregivers felt scared and nervous, as they had never cared for their child without the support of hospital staff.

Many families had to navigate a routine, via trial and error, which best suited their child’s needs and the family’s needs (22 in Table 3). Parent-caregivers cited having patience with themselves and their journeys as key to adjustment. Over time, parent-caregivers trusted themselves, their child, and other caregivers more. The complex tasks of tracheostomy care, ventilator alarms, medications, and therapy gradually settled into a consistent and predictable rhythm (23 in Table 3).

## 4. Discussion

The role of being a parent-caregiver of a tracheostomy-dependent child is composed of an intricate combination of medical and psychosocial domains. Understanding and appreciating the different challenges and roles that parent-caregivers need to embrace is crucial to effective collaboration between families and medical staff. This mixed-methods study explored the parent-caregiver perspective, identifying opportunities to better support parent-caregivers in tracheostomy education, training, and preparation for their child’s transition to home.

The message conveyed by our study was not that the tracheostomy itself created a change, but that the entire lived experience surrounding it created change in every aspect of the parent-caregivers’ lives. The evolution of roles between being a parent, caregiver, educator, employer, and entrepreneur (theme: *new identity formation*), as perceived and experienced by parent-caregivers, was a dynamic process. Not every parent-caregiver experienced this evolution in the same trajectory. Similar to nationally published data [28,29,30], most patients in our study had a tracheostomy placed in infancy, thus parent-caregivers learned parenting skills at the same time as tracheostomy skills (Table 1). Parent-caregivers initially focused on concrete tasks and skill building to attain the foundational knowledge to effectively care for their child. Over time, this expanded to completing more medically complex tasks, as well as parenting, teaching others, balancing family life, and championing their child’s wellbeing. This theme is supported in other studies, where parent-caregivers are described as undergoing a transformation in understanding their distinct roles as caregivers and parents [11,15,31]. For those parent-caregivers whose child received a tracheostomy later in life, learning the caregiving role may be a wholly different experience. One parent-caregiver commented on “not knowing your kid anymore” (4 in Table 3). This perceived distinction between parent and caregiver creates an internal role dichotomy with respect to the psychosocial and emotional components of parental identity [18]. Though there are two distinct components identified as part of the parent-caregiver role, those interviewed emphasized the importance of being considered a parent first and foremost. They expressed that being identified primarily as a caregiver diminished and dismissed an integral component of the relationship with their child (19 in Table 3).

In our study, parent-caregivers described feeling like nurses, which is consistent in other studies [11,32]. Due to medical complexity, tracheostomy-dependent children qualify for private duty nursing (PDN) services. PDN in the home aims to supplement, but not replace, the skilled nursing care that parent-caregivers provide their child [33]. However, the availability of private duty nurses who are skilled and experienced in pediatric tracheostomy care to provide family support can vary based on location, with those children residing in rural areas having very limited access to skilled nurses [32,34]. In addition, insurance companies closely review and may limit PDN hours per day [35]. These limitations impact parent-caregivers’ ability to fully utilize PDN as a resource, increasing the demands on their time, causing financial vulnerability and stress from taking time off from work, and potentially creating safety risks for the child if they are not able to identify a private duty nurse or another family member who may serve as a caregiver [36,37,38].

Because parent-caregivers understood that they were largely or solely responsible for their child’s medical care and wellbeing at home, they quickly realized that the training they received in the hospital was crucial for their child’s safety. There was significant overlap between the themes of *new identity formation* and *enduring education*. Entwined in the parent-caregiver transformation was the evolving learning environment. While they reported in survey data feeling “very” or “extremely” prepared to perform most tracheostomy skills at the time of their child’s transition from the hospital to home (Figure 3), interviews noted that their confidence to care for their child required development over time. In addition, while they reported their confidence in performing most tracheostomy skills at the time of this study, some still expressed feeling a lack of confidence to change tracheostomy tubes and ties, as well as recognize potential emergencies (Figure 2). Families shared that having an ongoing resource to review emergency care after discharge, including cardiopulmonary resuscitation, would have been valuable. However, some who sought community classes realized that they were not tailored to their medically complex child.

In addition, parent-caregivers in our study expressed a desire for more guidance and support around the less visible, behind-the-scenes challenges they faced. This included insurance regulations, PDN rules and paperwork, preparation of their home for their child and equipment, and the identification of local community resources to assist with home modifications, such as moving bedrooms, adding electrical circuitry, and installing accessible entryways. Parent-caregivers found social media outlets that provided tips and tricks for home life that hospital training did not cover, which included adapting the home, traveling, and managing PDN [35,39]. Parent-caregivers wished they could have anticipated or triaged these needs prior to going home, which emphasizes the importance of hospital staff working collectively as a team to help parent-caregivers prepare and anticipate how home life will likely change. Available guidance on preparing parent-caregivers for home is largely focused on medical needs and skills, rather than on practical needs and holistic support systems [13,35,40].

As hospital staff work with parent-caregivers, they need to be cognizant of individual learning and emotional needs to provide *psychosocial support* (theme). In our study, parent-caregivers approached this life change differently. Engaging the full multidisciplinary team can lessen parent-caregivers’ anxiety. For example, child life specialists can help parent-caregivers learn “new parent” information while they are learning the medical needs of their child [41]. Other specialists, such as occupational and physical therapists can help promote parent-child bonding by showing parent-caregivers how they may incorporate developmental care practices into everyday life. In addition, members of the hospital team can help parent-caregivers develop the confidence to safely hold their child in the presence of lines and devices [42].

As parent-caregivers engaged in ongoing learning and experienced a shift in identity, many emphasized the importance of having a strong support system. Our study mirrors others who have pointed to peer support as an additional way to support families [43]. Because the incidence of families caring for a child with a tracheostomy is low—roughly 6 per 100,000—finding support in the local community can be challenging [30]. Social media is a realistic outlet for caregivers to find other families who have had a similar journey [35]. Our institution has implemented a “Trach Buddy Program” to help families connect. The program was modeled from the Parent-to-Parent USA support program and the Canadian Premature Babies peer support model [44,45]. The main goal of peer support networks such as these is to partner families before tracheostomy tube placement and provide peer mentorship and support throughout their journey in the hospital and after discharge home [38,43]. Peer families also can provide ongoing support after tracheostomy-dependent children are discharged home as parent-caregivers navigate challenges and develop their own home routines as they work towards *establishing normalcy* (theme).

There is wide support for team-based, multidisciplinary, coordinated care of children with tracheostomies to prevent adverse outcomes. Varying levels of experience and confidence of medical staff can impact parent-caregiver education and long-term patient success and survival [46]. Medical staff may have limited exposure to the non-clinical setting and what is possible, and feasible, in the home environment. In the hospital, medical teams rely on information, real-time feedback, and constant measurement of outcomes. In the home setting, much of this is unknown. There is limited control over the home environment and a parent-caregiver’s actions and abilities. There is also limited knowledge about this experience, and how to help caregivers thrive in a less controlled and supported environment. The lack of standardized training, education, and simulation for medical staff and parent-caregivers highlights an opportunity to create an innovative educational program built on the needs of both medical staff and parent-caregivers [40,47]. Utilizing a team-based approach to education incorporates the inclusion of parent-caregivers in shared decision-making and participation in care [35,48].

### Study Limitations

The results of our study are limited to the experiences of parent-caregivers at a single center in Western New York, and the results may not be applicable to other areas of the United States. For instance, in our area, there is no long-term care facility within fifty miles able to manage children who are ventilator dependent, which greatly impacts discharge options for families. However, the findings of our study contribute to the growing body of the literature to better understand the experiences of families who have tracheostomy-dependent children. We excluded families whose primary language was not English, and those who did not have internet access, as our survey and interview process was performed virtually. Our survey was not designed to assess health literacy; however, we recognize the literature shows this is an important part of caregiver education. This is especially true for children with complex medical needs and elaborate discharge planning. Future studies focused on tailoring tracheostomy education to meet the health literacy needs of caregivers could improve caregiver competence and confidence during the discharge process [49,50]. We did not include a parent-caregiver as part of the core study team. However, one of the nurses on our study team provided her perspective and guidance on developing our survey and interview guide as a parent of a child with medical complexity. In addition, based on the parent-caregivers’ responses to interview questions, we added and modified questions during subsequent interviews to better capture the range of our parent-caregiver experiences. In addition, parent-caregivers may be incorporated as consultants or part of the research team, as there is some evidence that including patient engagement in quality improvement initiatives improves patient and provider satisfaction [51].

## 5. Conclusions

Children with tracheostomies represent a growing population, making it essential to explore the lived experiences of parent-caregivers who are directly involved in their care. Their insights can help hospital teams and policymakers guide the development of standardized education to prepare for their child’s transition home, as well as identify community and peer resources to support their ongoing psychosocial needs. Hospital staff and communities need to be cognizant of any limitations and barriers to providing consistent care in the home setting. Policymaker engagement is critical for supporting medically complex children. The current nursing workforce shortage, lack of long-term care options, and changes in state/federal aid, which hospitals cannot bridge alone, underscore this urgent need.

## Figures and Tables

**Table 1:** Demographic information of children whose parent-caregivers participated in this study.

Characteristics	Median (Q1, Q3) n = 15
Age (months) When obtained tracheostomy At time of study	4 (3, 6) 46 (30, 59)
Time in hospital (months) After obtained tracheostomy Total length of stay	3 (2, 6) 6 (5, 10)
Time at home, since hospital discharge (months)	42 (18, 54)

**Table 2:** Hospital location of tracheostomy and parent-caregiver education.

Characteristics	n (%) n = 15
Location at time of tracheostomy Neonatal intensive care unit Pediatric cardiac intensive care unit Pediatric intensive care unit	10 (67) 2 (13) 3 (20)
Location of caregiver education Neonatal intensive care unit Pediatric cardiac intensive care unit Pediatric intensive care unit Pediatric floor	6 (40) 4 (27) 4 (27) 1 (6)

**Table 3:** Themes and subthemes of parent-caregivers’ lived experiences.

Theme	Subtheme	Interview Excerpts
**New identity formation**	Caregiver	1. I just want to be a mom. I don’t want to have to be a nurse right now. I mean, basically we’re like mini LPNs—let’s just be honest. You know, that’s hard. 2. You don’t know what you don’t know. It’s your first time being home with your child, and it’s learning as you go.… 3. You aren’t living this life, so you don’t know what it’s like. When your baby is in the hospital, you can have time to sleep, you can have time to go home and shower, and you know when they come home, you’re not going to be able to do all those things normally.
	Educator	4. I don’t know what to tell her nurses. I think that’s the hardest part of coming home with this being brand new. You’re having to teach nurses about your child that you’re actually just learning… You don’t really know a lot about your kid anymore. You’re expected to teach somebody else about your kid and you’re like, “Okay, I can tell you what I found out the last five months that we’re going to learn together, buddy.” 5. A lot of the nurses that we had when we first came home, I had to train. I’ve had a lot of nurses say that they didn’t have experience working with kids on a ventilator, and they didn’t have experience working with the cough assist machine. So, that was a big gap. I’m fine with training new nurses and giving them the experience. I have a nurse that’s been with [my child] literally since the day he came home from the hospital. She didn’t know anything when she started with us. But now, she’s one of the most trusted nurses I have.
	Employer and Entrepreneur	6. You’re taking your medical fragile baby home. How would you also like to be the entrepreneur of a startup small company? Because now you’re going to have to manage inventory. You’re going to have to find, hire and fire nurses, and have employees, and just manage everything. And it’s a lot. 7. I was able to create a little small business within my house. It really took off. It’s been great, but not everybody can financially do that. It has been hard. I would not be able to hold a full-time job, and I probably wouldn’t even be able to hold a part-time job because you are relying on other people. We do have a phenomenal nursing schedule, but when people call-in [sick], then I have to call in. I can see that stress on a family. 8. We have so many filing systems. I have filing systems for her early intervention. I have filing systems for her medical. I have filing systems for her nursing.
**Enduring education**	Experiential learning	9. Really for us it was the emergency situations, like the scenarios that we might not have typically seen every day… and knowing what to do, because those scenarios can get very scary if we didn’t know. I think that was the best training—when you gave us those scenarios… 10. …A CPR class for parents would be awesome. We went and took one. Our city offers a free one thankfully, but there are a lot of cities who don’t offer them free. It came in handy with a lot of first aid tips, with resuscitation and learning the AED and all that stuff. We took our son and did a class with him, too. It’s something good for parents to have as a resource. 11. His dad and I watched YouTube every night. “Okay, we have to get ready. This is what we have to do.”
	Confidence development over time	12. It’s not going to be perfect. It’s not going to be easy. But I’ll get there. It’s just a rhythm you got to get into. Everybody has to find their own way. You can have all the training in the world, but until you’re really in it, it’s different. 13. I learned that I kind of freeze sometimes in emergencies, but I’m getting better at it as time’s gone by. You know sometimes you just have to do it and trust yourself that you know what to do… There have been times when she pulled her trach out, and I just put it back in. And there’s been times where she’s pulled it out when it’s just my husband. So, our confidence has gotten a lot higher.
	Parenting versus caregiving	14. On top of regular new mom baby stuff, now there’s stomas and all that kind of stuff. 15. I wish we had a little bit more on parenting. I mean, we are parenting… she’s not a typical child right? This really pushes… our parenting skills.
**Child and family biopsychosocial support**	Peer support (Child and Caregiver)	16. I know [getting a trach] is not something that happens every day at the hospital. I felt very scared and very alone… I’ve been buddied up with a trach family, and it was just nice to be able to have that support—to know what everyday life is like with a trach kid. It’s so important for families to be able to talk to someone that’s not in the medical field. 17. I think [my son] is always excited to see other kiddos who have trachs in place, especially when he sees a kiddo who is cognitively and socially where he is. He sees a kid with a trach, and he’s like, “Oh, this is so cool. Someone like me who I can chat with and have a conversation with and it doesn’t feel quite so different.” That’s I think wonderful.
	Compassion and respect	18. Be compassionate and patient, because it’s not your situation - it’s theirs. Everybody is not going to take to stuff right away. It can’t be a cookie-cutter, one-size-fits-all situation. It doesn’t work like that. Everybody has their own set of emotions, their own thought processes. It’s not going to be the same for everybody. 19. She’s a person... She’s more than special needs. She’s more than tubes and medicine…
**Establishing normalcy**	Simultaneous opposing emotions (fear, anxiety, excitement, relief)	20. I was very eager to get him home of course, because we’d been there for so long. It was just a scary situation. The first time when I was actually home with him -- and it was just me home with him -- I suddenly went, “Oh my God, I don’t have all the nurses who I would depend on.” I was doing a lot of his care in the NICU, but I always knew that there was always a nurse who’s just a shout away if anything went wrong. So, it was a scary moment to be like, “Oh, I’m really responsible for him now. 21. It’s amazing how uncomfortable you can feel in your own home... It’s getting better. It’s overwhelming at first, but it still is so much better to be home than in the hospital.
	Routine	22. You have this picture of walking in with your kid and expecting everything to kind of fall into place, and things aren’t always going to look the same way as they did in the hospital. It’s going to be different, but I think just be patient and really just focus on making sure that you know your kid’s safe and he has what he needs… Every single day is a different day. Some days, they’re really bad. Some days, they’re really stressful. Then there are other days when it’s amazing. Just taking it one day at a time and knowing that you just did the best job that you could for that day is important. 23. I think adjusting to the environment was our biggest thing. You know, you think, “Okay, I’m bringing my baby home. We’re going to walk through the doors, and we’re going to feel at ease.” And you know, it was stressful… Little things at home could really stress him out. He only knew the hospital room for so long. Then you come home, and you have pets and family and all new lights and all those things that you really wouldn’t think would affect a child, but it really did. It really did. Home life definitely changed, but we got into a rhythm. It’s amazing, and he’s thriving now.

## Data Availability

The raw data supporting the conclusions of this article will be made available by the authors upon request.

## Appendix A

**Table A1:** Parent-Caregiver Survey.

*For the following questions, confidence is defined as the feeling or belief that you can perform the skill well*. *Not at all confident: I want to watch someone else perform the skill* *Slightly confident: I feel comfortable with another caregiver assisting 100% of the skill* *Moderately confident: I can perform skill with assistance 50% of the time interested in utilizing* *Quite confident: I can perform skill independently, may need to ask for help or reference training materials* *Very confident: I feel comfortable performing the skill independently* *Extremely confident: I feel comfortable performing the skill independently, and can teach the skill to others* *Please rate your level of confidence for the following tracheostomy skills below*						
	Not at all confident	Slightly confident	Moderately confident	Quite confident	Very confident	Extremely confident
Changing tracheostomy ties	○	○	○	○	○	○
Changing the tracheostomy dressing	○	○	○	○	○	○
Recognize signs that could indicate a tracheal stoma infection	○	○	○	○	○	○
Recognize warning signs of respiratory distress	○	○	○	○	○	○
Identifying when my child needs to be suctioned	○	○	○	○	○	○
My ability to correctly operate suction catheter	○	○	○	○	○	○
My ability to calculate the suctioning depth	○	○	○	○	○	○
Placing a pulse oximeter and managing the alarms	○	○	○	○	○	○
Recognize and respond to a trach dislodgment	○	○	○	○	○	○
Using a home ventilator such as LTV or Trilogy	○	○	○	○	○	○
My ability to transport my child in stroller, wheelchair, car, etc.	○	○	○	○	○	○
My ability to assess if my child needs urgent medical attention (call 911, go to Emergency Department)	○	○	○	○	○	○
How well did the education you received in the hospital prior to discharge prepare you for the following skills when you went home? Not at all prepared: *I did not feel comfortable performing this skill* Slightly prepared: *I could not perform skill independently and needed a second caregiver assisting me with the skill* Somewhat prepared: *I could perform the skill but preferred that a second caregiver be available as a resource* Quite prepared: *I was confident performing the skill independently, I would like a second caregiver available as back-up* Very prepared: *I was confident performing the skill independently, and am confident in being alone with my child* Extremely prepared: *I was confident being alone with my child and felt confident training other caregivers in the care of my child*						
	Not at all confident	Slightly confident	Moderately confident	Quite confident	Very confident	Extremely confident
Changing tracheostomy ties	○	○	○	○	○	○
Changing the tracheostomy dressing	○	○	○	○	○	○
Changing the tracheostomy tube	○	○	○	○	○	○
Recognize signs that could indicate a tracheal stoma infection	○	○	○	○	○	○
Recognize warning signs of respiratory distress	○	○	○	○	○	○
Identifying when my child needs to be suctioned	○	○	○	○	○	○
My ability to correctly operate suction catheter	○	○	○	○	○	○
My ability to calculate the suctioning depth	○	○	○	○	○	○
Placing a pulse oximeter and managing the alarms	○	○	○	○	○	○
Recognize and respond to a trach dislodgment	○	○	○	○	○	○
Using a home ventilator such as LTV or Trilogy	○	○	○	○	○	○
My ability to transport my child in stroller, wheelchair, car, etc.	○	○	○	○	○	○
My ability to assess if my child needs urgent medical attention (call 911, go to Emergency Department)	○	○	○	○	○	○
Can you elaborate on situations where you would contact the Ears Nose Throat (ENT) team versus when you would contact the Pulmonary Team (lung team).						
The person I would contact if I needed help training a new caregiver in the care of my child’s trach would be...						
Please elaborate on any concerns that you have when thinking about caring for your child at home.						
Was there anything that you were surprised about when caring for your child at home?						
Are there any other topics you would have liked education on?						
Having an outpatient option to train additional caregivers (who were not trained as part of the initial hospitalization) is a service that I would be interested in utilizing?	○Yes ○No					
Would you be interested in an individual interview where you could talk more about your teaching experience in the hospital and how that prepared you and your family for taking care of your child at home?	○Yes ○No					

**Table A2:** Parent-Caregiver Interview Guide.

	Interview Question
1.	How many children with medical needs do you care for?
2.	What are the medical needs of the child/children? A. What type of medical equipment do you use in the home? B. Do you know who to contact if there are issues with the home equipment?
3.	How long ago was the first discharge home from the hospital?
4.	What training did you receive in the hospital to prepare you for discharge home? Who completed training with you?
5.	Do you feel like you were adequately trained to take your child home from the hospital? Why or why not?
6.	What training do you wish you had received prior to discharge from the hospital, if any?
7.	What additional practice would have been helpful prior to discharge, if any?
8.	What specific training was most helpful to you prior to discharge?
9.	What training was least helpful to you prior to discharge?
10.	Have you experienced any medical emergencies at home with your child? A. If so, what was the emergency? B. At the time, did you feel prepared to handle the emergency? Why or why not? C. Did an ambulance/EMS need to be called?
11.	On a scale of 1–10 (1 being not making a difference at all, 10 making a significant difference) how much do you think training with a realistic mannequin would have made you feel more prepared for discharge? A. What makes you choose that number?
12.	What resources do you use at home to help care for your child? Are there any other resources you wish you had available to you, such as a GCH-specific website, care binder, etc?
13.	Was there anything that surprised you about your child’s care when you got home?
14.	What advice would you give other caregivers preparing to take their child home from the hospital for the first time?
15.	What advice would you give staff at the hospital teaching parents of medically complex children?
16.	What kind of help do you have in the home? A. Does the current help and structure allow you to be able to handle caring for your child? B. What additional supports in the home would be beneficial? C. Have you ever used respite services? Would you be interested in respite services?

## References

[1] Sherman J., Zalzal H., Bower K. (2024). Equitable Care for Children With a Tracheostomy: Addressing Challenges and Seeking Systemic Solutions. Health Expect.

[2] Kuo D.Z., Houtrow A.J., Disabilities C.O.C.W. (2016). Recognition and Management of Medical Complexity. Pediatrics.

[3] Kremer B., Botos-Kremer A.I., Eckel H.E., Schlöndorff G. (2002). Indications, complications, and surgical techniques for pediatric tracheostomies—An update. J. Pediatr. Surg..

[4] Roberts J., Powell J., Begbie J., Siou G., McLarnon C., Welch A., McKean M., Thomas M., Ebdon A., Moss S. (2020). Pediatric tracheostomy: A large single-center experience. Laryngoscope.

[5] Fuller C., Wineland A.M., Richter G.T. (2021). Update on Pediatric Tracheostomy: Indications, Technique, Education, and Decannulation. Curr. Otorhinolaryngol. Rep..

[6] Spratling R. (2017). Understanding the health care utilization of children who require medical technology: A descriptive study of children who require tracheostomies. Appl. Nurs. Res..

[7] Mitchell R.B., Hussey H.M., Setzen G., Jacobs I.N., Nussenbaum B., Dawson C., Brown C.A., Brandt C., Deakins K., Hartnick C. (2013). Clinical Consensus Statement: Tracheostomy Care. Otolaryngol. Head Neck Surg..

[8] Wells S., Shermont H., Hockman G., Hamilton S., Abecassis L., Blanchette S., Munhall D. (2018). Standardized Tracheostomy Education Across the Enterprise. J. Pediatr. Nurs..

[9] Tolomeo C., Major N.E., Szondy M.V., Bazzy-Asaad A. (2017). Standardizing Care and Parental Training to Improve Training Duration, Referral Frequency, and Length of Stay: Our Quality Improvement Project Experience. J. Pediatr. Nurs..

[10] Nageswaran S., Golden S.L., Gower W.A., King N.M. (2018). Caregiver Perceptions about their Decision to Pursue Tracheostomy for Children with Medical Complexity. J. Pediatr..

[11] Acorda D.E., Jackson A., Lam A.K., Molchen W. (2022). Overwhelmed to ownership: The lived experience of parents learning to become caregivers of children with tracheostomies. Int. J. Pediatr. Otorhinolaryngol..

[12] Weir M., Kubba H. (2025). Psychological Challenges in Children with Tracheostomies and Their Families—A Qualitative Study. Clin. Otolaryngol..

[13] Desai A.D., Durkin L.K., Jacob-Files E.A., Mangione-Smith R. (2016). Caregiver Perceptions of Hospital to Home Transitions According to Medical Complexity: A Qualitative Study. Acad. Pediatr..

[14] Antoniou I., Wray J., Kenny M., Hewitt R., Hall A., Cooke J. (2022). Hospital training and preparedness of parents and carers in paediatric tracheostomy care: A mixed methods study. Int. J. Pediatr. Otorhinolaryngol..

[15] Van Orne J.A., Clutter P., Fredland N., Schultz R. (2024). Caring for the child with a tracheostomy through the eyes of their caregiver: A photovoice study. J. Pediatr. Nurs..

[16] Ramanathan D., Bruckman D., Appachi S., Hopkins B. (2024). Association of Discharge Location Following Pediatric Tracheostomy with Social Determinants of Health: A National Analysis. Otolaryngol. Head Neck Surg..

[17] Cecil C.A., Dziorny A.C., Hall M., Kane J.M., Kohne J., Olszewski A.E., Rogerson C.M., Slain K.N., Toomey V., Goodman D.M. (2024). Low-Resource Hospital Days for Children Following New Tracheostomy. Pediatrics.

[18] Crocker A.F., Smith S.N. (2019). Person-first language: Are we practicing what we preach?. J. Multidiscip. Healthc..

[19] Rickards G., Magee C., Artino A.R. (2012). You Can’t Fix by Analysis What You’ve Spoiled by Design: Developing Survey Instruments and Collecting Validity Evidence. J. Grad. Med. Educ..

[20] Harris P.A., Taylor R., Minor B.L., Elliott V., Fernandez M., O’Neal L., McLeod L., Delacqua G., Delacqua F., Kirby J. (2019). The REDCap Consortium: Building an international community of software platform partners. J. Biomed. Inform..

[21] E.S. Corporation MyChart. https://www.epic.com/.

[22] Z. Communications Zoom. https://www.zoom.com/.

[23] R. Communications https://www.rev.com/.

[24] Charmaz K. (2015). Teaching Theory Construction with Initial Grounded Theory Tools: A Reflection on Lessons and Learning. Qual. Health Res..

[25] Merriam S.B., Elizabeth J. (2016). Qualitative Research. A Guide to Design and Implementation.

[26] Verdinelli S., Scagnoli N.I. (2013). Data Display in Qualitative Research. Int. J. Qual. Methods.

[27] Borkan J.M. (2022). Immersion–Crystallization: A valuable analytic tool for healthcare research. Fam. Pr..

[28] Brown C., Shah G.B., Mitchell R.B., Lenes-Voit F., Johnson R.F. (2021). The Incidence of Pediatric Tracheostomy and Its Association Among Black Children. Otolaryngol. Head Neck Surg..

[29] Hebbar K.B., Kasi A.S., Vielkind M., McCracken C.E., Ivie C.C., Prickett K.K., Simon D.M. (2021). Mortality and Outcomes of Pediatric Tracheostomy Dependent Patients. Front. Pediatr..

[30] Muller R.G., Mamidala M.P., Smith S.H., Smith A., Sheyn A. (2019). Incidence, Epidemiology, and Outcomes of Pediatric Tracheostomy in the United States from 2000 to 2012. Otolaryngol. Head Neck Surg..

[31] Koch A., Kozhumam A.S., Seeler E., Docherty S.L., Brandon D. (2021). Multiple Roles of Parental Caregivers of Children with Complex Life-Threatening Conditions: A Qualitative Descriptive Analysis. J. Pediatr. Nurs..

[32] Sobotka S.A., Dholakia A., Berry J.G., Brenner M., Graham R.J., Goodman D.M., Agrawal R.K. (2020). Home nursing for children with home mechanical ventilation in the United States: Key informant perspectives. Pediatr. Pulmonol..

[33] N.Y. State (2025). Private Duty Nursing Policy Manual.

[34] Juraschek S.P., Zhang X., Ranganathan V., Lin V.W. (2019). Republished: United States Registered Nurse Workforce Report Card and Shortage Forecast. Am. J. Med. Qual..

[35] Amar-Dolan L.G., Horn M.H., O’cOnnell B., Parsons S.K., Roussin C.J., Weinstock P.H., Graham R.J. (2020). “This Is How Hard It Is”. Family Experience of Hospital-to-Home Transition with a Tracheostomy. Ann. Am. Thorac. Soc..

[36] Baddour K., Mady L.J., Schwarzbach H.L., Sabik L.M., Thomas T.H., McCoy J.L., Tobey A. (2021). Exploring caregiver burden and financial toxicity in caregivers of tracheostomy-dependent children. Int. J. Pediatr. Otorhinolaryngol..

[37] Foster C.C., Chorniy A., Kwon S., Kan K., Heard-Garris N., Davis M.M.M. (2021). Children with Special Health Care Needs and Forgone Family Employment. Pediatrics.

[38] Truitt B.A., Ghosh R.N., Price E.W., Du C., Bai S., Greene D., Simon D.M., Reeder W., Kasi A.S. (2025). Family Caregiver Knowledge in the Outpatient Management of Pediatric Tracheostomy-Related Emergencies. Clin. Pediatr..

[39] Huestis M.J., Kahn C.I., Tracy L.F., Levi J.R. (2020). Facebook Group Use among Parents of Children with Tracheostomy. Otolaryngol. Head Neck Surg..

[40] Acorda D.E., Van Orne J. (2025). Pediatric Tracheostomy Education Program Structures, Barriers, and Support: A Nationwide Survey of Children’s Hospitals. Otolaryngol. Head. Neck Surg..

[41] Basak R.B., Momaya R., Guo J., Rathi P. (2019). Role of Child Life Specialists in Pediatric Palliative Care. J. Pain. Symptom Manag..

[42] Dudek-Shriber L. (2004). Parent Stress in the Neonatal Intensive Care Unit and the Influence of Parent and Infant Characteristics. Am. J. Occup. Ther..

[43] Shilling V., Morris C., Thompson-Coon J., Ukoumunne O., Rogers M., Logan S. (2013). Peer support for parents of children with chronic disabling conditions: A systematic review of quantitative and qualitative studies. Dev. Med. Child. Neurol..

[44] Parent to Parent USA https://www.p2pusa.org/.

[45] CPBF Canadian Premature Babies Foundation (2025). https://www.cpbf-fbpc.org/.

[46] McKeon M., Kohn J., Munhall D., Wells S., Blanchette S., Santiago R., Graham R., Nuss R., Rahbar R., Volk M. (2019). Association of a Multidisciplinary Care Approach with the Quality of Care After Pediatric Tracheostomy. JAMA Otolaryngol. Head Neck Surg..

[47] Abode K.A., Drake A.F., Zdanski C.J., Retsch-Bogart G.Z., Gee A.B., Noah T.L. (2016). A Multidisciplinary Children’s Airway Center: Impact on the Care of Patients With Tracheostomy. Pediatrics.

[48] Franck L.S., Axelin A., Van Veenendaal N.R., Bacchini F. (2023). Improving Neonatal Intensive Care Unit Quality and Safety with Family-Centered Care. Clin. Perinatol..

[49] Glick A.F., Farkas J.S., Mendelsohn A.L., Fierman A.H., Tomopoulos S., Rosenberg R.E., Dreyer B.P., Melgar J., Varriano J., Yin H.S. (2019). Discharge Instruction Comprehension and Adherence Errors: Interrelationship Between Plan Complexity and Parent Health Literacy. J. Pediatr..

[50] Keim-Malpass J., Letzkus L.C., Kennedy C. (2015). Parent/caregiver health literacy among children with special health care needs: A systematic review of the literature. BMC Pediatr..

[51] Marzban S., Najafi M., Agolli A., Ashrafi E. (2022). Impact of Patient Engagement on Healthcare Quality: A Scoping Review. J. Patient Exp..

